# Resuscitative Thoracotomy for Multiple Gunshot Wounds With Cardiac Tamponade Despite Pericardial Window

**DOI:** 10.7759/cureus.11907

**Published:** 2020-12-04

**Authors:** Nathan Kostick, Sanjiv Gray, Dustin Huynh

**Affiliations:** 1 Medicine, University of Central Florida College of Medicine, Orlando, USA; 2 Surgery, University of Central Florida College of Medicine, Orlando, USA; 3 Trauma and Acute Care Surgery, Osceola Regional Medical Center, Kissimmee, USA

**Keywords:** trauma, thoracotomy, emergency medicine, thoracic surgery, gsw, tamponade, coagulopathy of trauma

## Abstract

This report reviews the indications and complications of resuscitative thoracotomy in the trauma patient as seen with the clinical course of a 19-year-old male who experienced postoperative pericardial tamponade after a bilateral resuscitative thoracotomy with pericardiotomy. This patient presented to the hospital in critical condition with 31 gunshot wounds (GSWs) distributed over the chest, abdomen, and extremities. After undergoing an initially successful resuscitative thoracotomy, the patient continued to bleed into his chest at a greater rate than the chest tubes were able to adequately evacuate. Despite the presence of a large pericardial window, clotted blood led to cardiac tamponade. Subsequent bedside reopening of thoracotomy under conscious sedation (ketamine, fentanyl, and midazolam) was required to evacuate the clots and stabilize the patient. This case provides the opportunity to discuss several interesting points for managing the traumatized patient, including indications for resuscitative thoracotomy, use of conscious sedation for bedside major surgery, and complications of clamshell thoracotomy, and ethics of resource allocation.

## Introduction

Clamshell thoracotomy or bilateral resuscitative thoracotomy is a last-resort life-saving procedure for patients with severe thoracic trauma. The goal of this therapy is frequently gain rapid access to the heart or major thoracic vessels to control bleeding and tamponade [1-5]. Resuscitative thoracotomy has a dismal survival rate of 7.4% [5, 6]. However, those who survive tend to have good outcomes, with up to 86% leaving neurologically intact and 75% returning to normal activities [7, 8]. It is important to note that the survival rates differ greatly based on injury mechanism - those with blunt trauma have significantly worse outcomes than penetrating injury (1.6% vs. 11.2% survival) [5, 9, 10]. The clamshell thoracotomy is performed by making an incision at the fifth intercostal space bilaterally from the midaxillary line to the sternum. The sternum is then cut with a Gigli saw and sternal retractors placed horizontally to gain an unobstructed view of the bilateral lung fields, great vessels, and pericardium [11]. Of note, a review of the literature failed to find a single case report of cardiac tamponade with an open pericardial window, as seen in our patient.

## Case presentation

**Figure 1:**
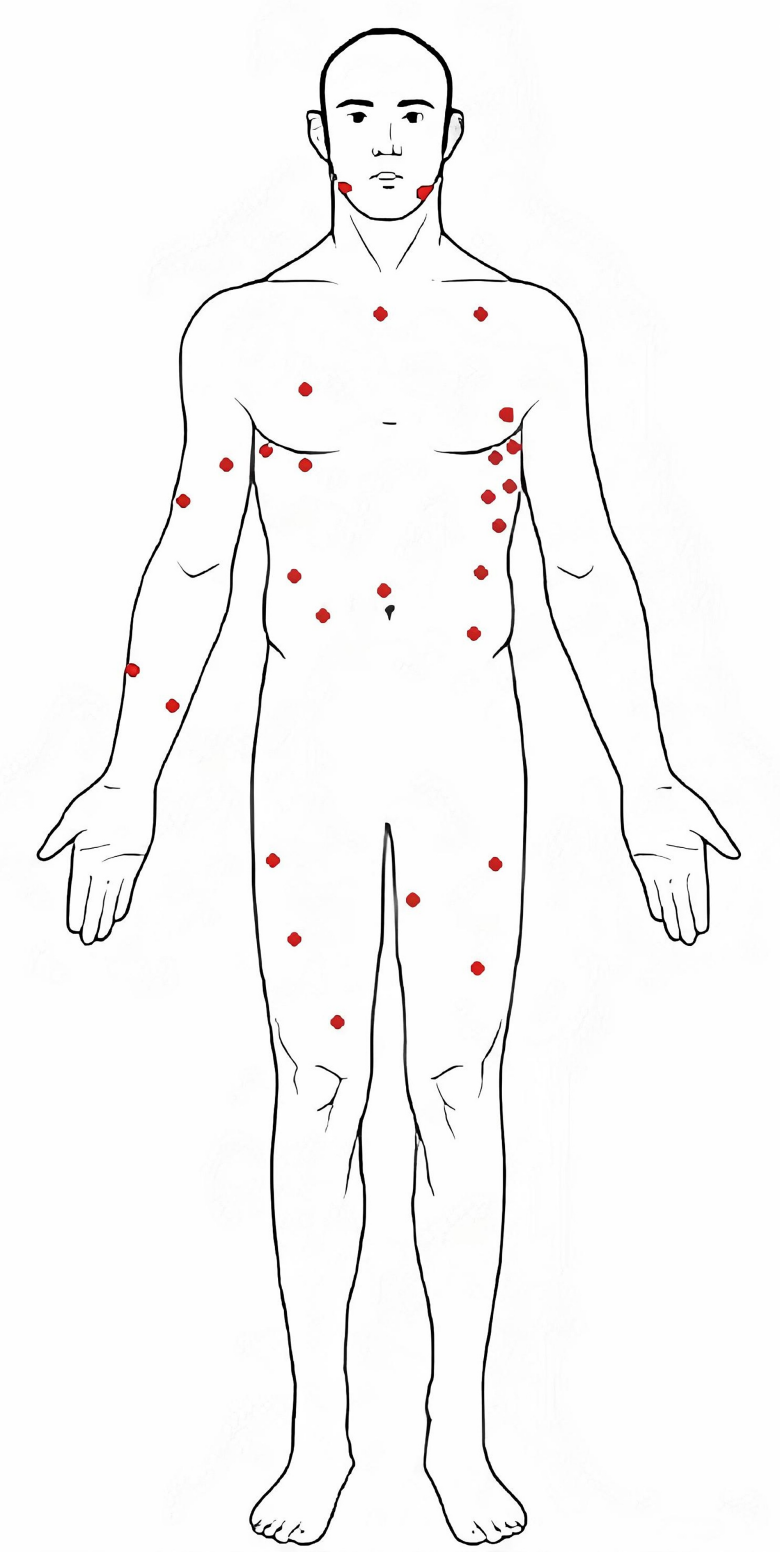
Distribution of gunshot wounds Each red mark represents the approximate location of the gunshot wound.

A 19-year-old male presented to a Level II trauma center by emergency medical services (EMS)in hemorrhagic shock with multiple gunshot wounds (GSWs). EMS performed a "scoop and run" and provided fluid support via normal saline bolus during transport.AMPLE (allergies, medications, past medical history, last eaten, events leading)history was not available to EMS due to the patient’s injuries. Upon arrival, he was unresponsive with vitals of blood pressure 80/50 mmHg, heart rate 120, oxygen saturation (SpO2) 92% with a laryngeal airway. A total of 31 gunshot wounds were found on the patient’s body in the distribution seen in Figure 1. The patient was unable to be rolled his back examined for additional wounds due to his critical state.

The primary survey was performed in a "circulation, airway, breathing" (CAB) fashion due to multiple high energy penetrating trauma to the chest and abdomen.

Circulation: The patient demonstrated hemorrhagic shock with hypotension and tachycardia. Mass transfusion protocol was activated, and two units of uncross-matched whole red blood were given immediately by pressure infusion. Evidence of hemorrhage was present in three out of the five fatal fields: chest (breathing), abdomen (distended and firm), and floor. Bleeding from the extremity GSWs were relatively mild and non-pulsatile. Suspicion of pelvic hemorrhage was low with the GSW’s sparing this region (later confirmed intraoperatively as well as with adjuvant surveys).

Airway: Rapid sequence intubation was performed to secure the airway, owing to the patient's altered mental status and evidence of multiple penetrating trauma to the jaw and neck (three bullet wounds present - two submandibular, one in the right buccal region).

Breathing: The patient was breathing spontaneously prior to rapid sequence intubation (RSI); however, breath sounds were diminished bilaterally. Bilateral chest tubes were placed, yielding greater than 1L of blood loss from the left chest tube and 500mL from the right chest tube. Subsequently, a left ED thoracotomy was performed to control bleeding. Bleeding was initially controlled through ligation of the internal mammillary artery, an intercostal artery, and branches of the pulmonary artery, as well as packing of the wound.

**Figure 2:**
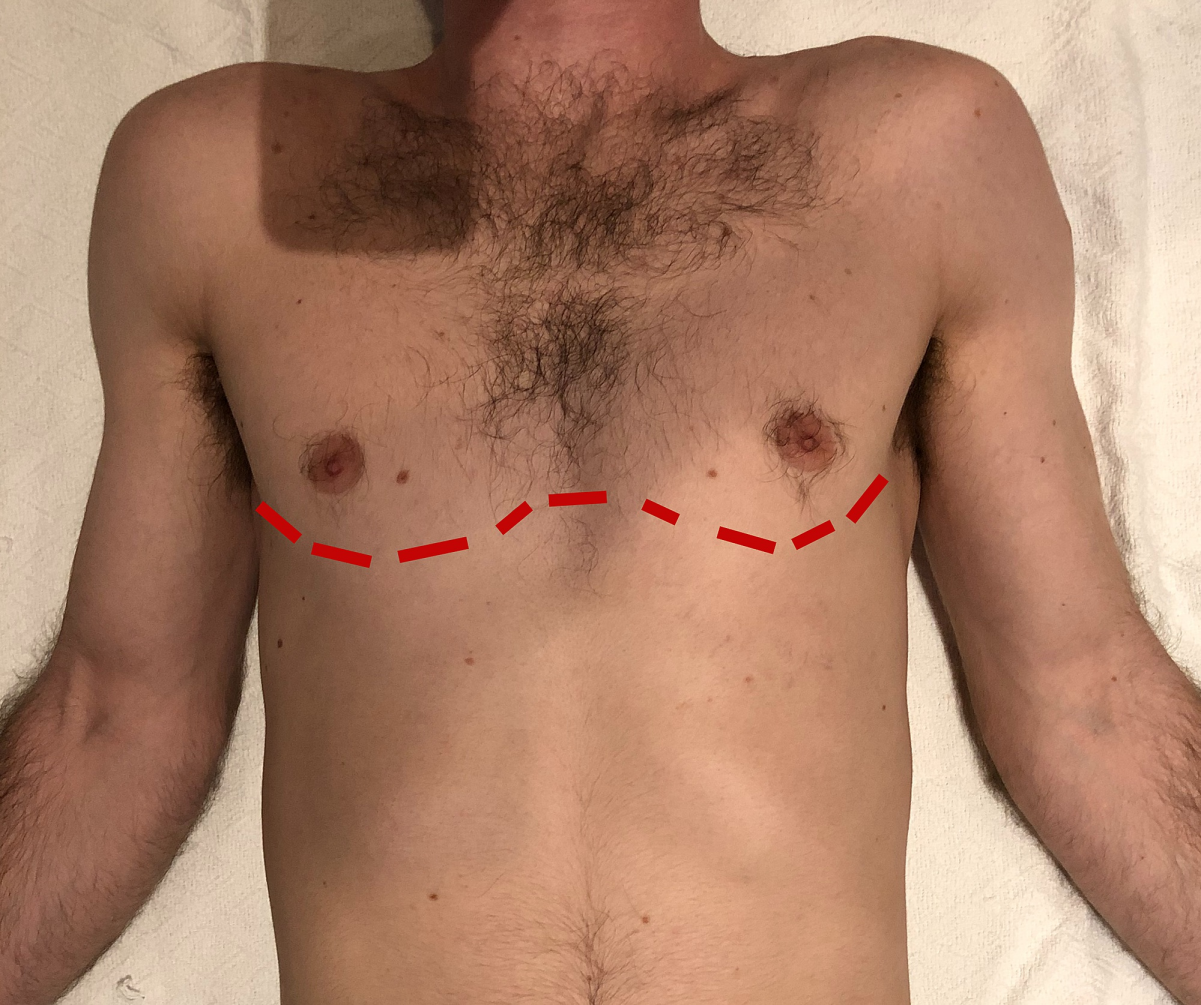
Approximate incision site for clamshell thoracotomy

After two additional units of uncross-matched universal whole blood and initial control of hemorrhage, the patient was stable enough for transfer to the operating room (OR). The patient was prepared for emergent exploratory laparotomy with damage control surgery. Significant bleeding and fecal contamination was found with injuries to the liver, colon, small bowel, spleen, and left iliac artery. Hemicolectomy, small bowel resection, splenectomy, and iliac artery laceration repair was required. Splinting was required for the right upper extremity fractures. During the course of the damage control surgery, over 1L of blood was evacuated via the left chest tube, and the left ED thoracotomy was extended to a bilateral clamshell thoracotomy (approximate incision site can be seen in Figure 2). Further control of hemorrhage was controlled with ligation of the internal mammillary arteries, packing, and right middle lobectomy. A large pericardial window was also performed to evacuate a clot and prevent further tamponade. The patient's thoracotomy was temporarily closed with sutures, and the abdomen was left open with Abthera™ wound vacuum-assisted closure (VAC) placement. Throughout the course of this operation, an additional 16 units of packed red blood cells (pRBCs), 11 platelets, and eight fresh frozen plasma (FFP) were transfused with supportive calcium replenishment. After major hemorrhage was controlled in both the chest and abdomen, the patient was transferred directly to the intensive care unit (ICU) with direct physician supervision. Rotational thromboelastometry (ROTEM) and other laboratory studies were used to guide the intraoperative and postoperative resuscitation.

Over the next hour, the patient continued to be intermittently hypotensive to 40 mmHg mean arterial pressure (MAP) but would respond to blood products up to 85 mmHg MAP. During this time, the patient continued to put out fluid from his bilateral chest tube 300ml from the right tube, 500mL from the left. Despite this, the patient was responsive and was able to answer a series of "yes/no" questions by shaking his head (Glasgow Coma Scale [GCS] 11T). The patient progressively desaturated to 80% SpO2 despite adequate adjustment of the ventilatory settings. Bedside bronchoscopy with suction and lavage was able to return the patient's SpO2 to >90%. In this immediate postoperative period, an additional nine units of pRBCs, six platelets, and six FFP were given. However, the patient continued to decompensate without frequent transfusions. Surprisingly, bleeding studies (ROTEM) returned a normocoagulative state. An additional 1L of blood had been drained from the left chest tube, 400mL from the right chest tube, and 300 mL from the abdominal wound VAC. Additionally, the pulse pressure had been progressively diminishing during this time. Bedside echocardiogram showed fluid surrounding the heart, with evidence of right ventricular collapse despite having a pericardial window. Bedside reopening of the thoracotomy was performed under procedural sedation with a combination of ketamine, fentanyl, and midazolam. The reopening of the chest found active pulsatile bleeding from the left internal mammillary artery as well as significant clotted blood throughout the chest, including surrounding the heart. The mammillary artery was ligated, the pericardial window was further extended, and the clots evacuated from the chest. This resulted in the stabilization of the patient's vitals. Additional chest tubes and pericardial drains were placed. Over the next 12 hours, the patient was relatively stable, requiring ventilatory support and occasional transfusion. However, the patient suffered cardiac arrest and rapidly decompensated. The patient’s chest and abdomen were reopened, showing clotted hemothorax, hemopericardium, and ischemic bowel. The patient suffered a severe anoxic brain injury (GCS 3) and ultimately succumbed to his injuries despite aggressive resuscitation. Interestingly, pathology discovered pulmonary artery occlusion by liver tissue emboli, which likely contributed to the patient's cardiac arrest and disseminated intravascular coagulation (DIC).

## Discussion

This paper highlights indications for ED thoracotomy and clamshell thoracotomy, complications of clamshell thoracotomy, use of procedural sedation for bedside thoracotomy, and ethical considerations in resource utilization.

First, a brief review of the indications and contra-indications of the resuscitative thoracotomy. The indications for resuscitative thoracotomy include thoracic trauma (blunt or penetrating) with hemodynamic instability that does not respond to fluid resuscitation and shock that is correctable by specific surgical maneuvers such as cross-clamping the aorta, pericardial fenestration, direct cardiovascular repair, and evacuation of air embolism. Contraindications to resuscitative thoracotomy are primarily limited to unsurvivable situations such as asystole without pericardial tamponade, prolonged pulselessness, ordead on arrival [DOA]/non-survivable injuries [1, 5, 8]. Our patient met the indications for resuscitative. He was young, responsive in the field but in critical condition after penetrating trauma to the chest, required pericardial fenestration and vascular repair, and did not demonstrate any contraindication to resuscitative thoracotomy. The strength of this procedure is supported by our patient's initial stabilization of his critical state to the point of cooperation. 

Second, clamshell thoracotomy complications include infection, transection of intercostal and internal mammillary arteries, fracture of ribs, and damage to the phrenic nerves. The risk of complications is increased due to the fact that the resuscitative thoracotomy is frequently performed under non-ideal situations, such as in the trauma bay (vs. OR), on unstable patients, and a paucity of cases performed [4, 5]. Our patient experienced the relatively common complication bleeding as well as the rare complication of cardiac tamponade despite the pericardial window. A literature review failed to find a report of early recurrence of cardiac tamponade in a traumatized patient with a pericardial window. It is unclear whether our patient's persistent intrathoracic bleeding which led to the tamponade, was secondary to his initial injuries, the clamshell thoracotomy, or a combination of both. In the situation of continued hemodynamic instability without an obvious cause, one must exclude coagulopathy, missed injury, and non-functioning drains (as seen in our patient).

Third, although the use of ketamine, fentanyl, and/or midazolam for procedural sedation is common practice for many procedures such as endoscopy or chest tube insertion, its use for major surgery in the traumatized patient has not been well described [12, 13]. This combination of medications is ideal for the traumatized patient with their lack of cardiovascular depressant effects and minimal concern for medication-induced respiratory depression since these patients are already mechanically ventilated. This technique can be used with induction dosing followed by a maintenance drip or through PRN boluses. Our team performed the bedside reopening of the clamshell thoracotomy without interruptions from hypoalgesia or anesthetic related hemodynamic instability.

Resource allocation for severely traumatized patients such as ours is always challenging. Our patient required over 40 units of pRBC, 35 of FFP, and 35 platelets reduced the supply available for other patients. In addition to the non-surgical teams such as anesthesia, pharmacy, nursing, and blood bank, this patient required five hours in the OR for initial damage control with three attending surgeons, two residents, and a physician assistant (PA)**, **followed by two bedside reopenings of the thoracotomy and nearly 24 hours of direct physician attendance in the ICU. This is a significant investment of resources for a hospital of any size. The just and equitable resource distribution was discussed during the care of this patient. However, based on our patient's initial response to intervention, we decided to continue heroic efforts. Despite unprecedented efforts, the patient ultimately succumbed to his injuries. Ultimately, it would have been unethical to discontinue efforts due to the patient's intact neurological exam and initial ability to respond to questions.

## Conclusions

This case provided an opportunity to review several important considerations in managing major penetrating trauma to the thorax, including the indications for ED thoracotomy and clamshell thoracotomy, the clamshell complications thoracotomy, the use of procedural sedation for bedside thoracotomy, and ethical considerations in resource utilization. Additionally, we discussed the unusual presentation of cardiac tamponade despite the presence of a pericardial window. Surgeons should consider the presence of clotted hemopericardium in patients who are showing signs of obstructive cardiac failure despite appropriate treatment (pericardial window and drains).
